# Redox Role of *Lactobacillus casei* Shirota Against the Cellular Damage Induced by 2,2′-Azobis (2-Amidinopropane) Dihydrochloride-Induced Oxidative and Inflammatory Stress in Enterocytes-Like Epithelial Cells

**DOI:** 10.3389/fimmu.2018.01131

**Published:** 2018-05-24

**Authors:** Alberto Finamore, Roberto Ambra, Fabio Nobili, Ivana Garaguso, Anna Raguzzini, Mauro Serafini

**Affiliations:** ^1^Centre for Food and Nutrition, Council for Agricultural Research and Economics, Rome, Italy; ^2^Functional Foods and Metabolic Stress Prevention Laboratory, Faculty of Bioscience and Technology for Food, Agriculture and Environment, University of Teramo, Teramo, Italy

**Keywords:** oxidative stress, probiotics, *Lactobacillus* shirota, antioxidants, human colon carcinoma cell line, inflammation, nuclear factor kappa B, nuclear factor erythroid 2-related factor 2

## Abstract

In western societies where most of the day is spent in the postprandial state, the existence of oxidative and inflammatory stress conditions makes postprandial stress an important factor involved in the development of cardiovascular risk factors. A large body of evidence have been accumulated on the anti-inflammatory effects of probiotics, but no information is available on the mechanisms through which intestinal microbiota modulates redox unbalance associated with inflammatory stress. Here, we aimed to investigate the ability of *Lactobacillus casei* Shirota (LS) to induce an antioxidant response to counteract oxidative and inflammatory stress in an *in vitro* model of enterocytes. Our results show that pretreatment of enterocytes with LS prevents membrane barrier disruption and cellular reactive oxygen species (ROS) accumulation inside the cells, modulates the expression of the gastro-intestinal glutathione peroxidase (GPX2) antioxidant enzyme, and reduces p65 phosphorylation, supporting the involvement of the Nfr2 and nuclear factor kappa B pathways in the activation of antioxidant cellular defenses by probiotics. These results suggest, for the first time, a redox mechanism by LS in protecting intestinal cells from AAPH-induced oxidative and inflammatory stress.

## Introduction

Probiotics have already proven their beneficial effects in the treatment of several intestinal inflammatory pathologies and in their ability to modulate the immune response and protect the intestinal epithelial barrier (1–3). Stimulation of the intestine with bacterial preparations has been shown to specifically reduce pro-inflammatory cytokines production and enhance *ex vivo* synthesis of IL-4 and IL-10 by CD4^+^ T cells. Using *in vitro* systems, immune and anti-inflammatory health effects of probiotics in both human and animal hosts were shown to be strain dependent (4) i.e. very high in the *Lactobacilli genus* (5), and associated with peculiar antioxidant capacities (6). However, no information is available about the redox response during the anti-inflammatory effect of probiotics. A recent *in vivo* study shows that the supplementation of *Lactobacillus rhamnosus* reduces plasma and liver oxidative stress induced by a high-fat diet (HFD) in diabetic rats through a modulation of catalase and glutathione peroxidase (GPx) activities (7).

Normally, GPx and catalase mitigate oxidative damages and maintain the cellular redox homeostasis thanks to a tight regulation by nuclear factor (erythroid-derived 2)-like2 (Nrf2), that is sensitive and reacts to inflammatory events induced by reactive oxygen species (ROS) (8, 9). ROS are able to activate the nuclear factor kappa B (NF-κB) cascade (10–12), leading to the production of pro-inflammatory molecules, including cytokines and chemokines (13). Antagonism and synergy occur between members of these two pathways through direct effects on transcription factors, protein–protein interactions, or second-messenger effects on target genes. However, chronic inflammatory or oxidative stresses, for example, upon inadequate food intake, are well-known pathogenetic risk factors for obesity, CVD, diabetes, and cancer (14, 15).

The instauration of food-related chronic oxidative and inflammatory stresses is actually very common in Western societies (16), where most of the day is spent in the postprandial status and high-fat meals (HFM) are consumed. The inflammatory response induced by HFM, from one hand is mediated by pro-inflammatory cytokines, glycemia/insulin response, and oxidized lipids (17), from the other hand triggers an endogenous antioxidant response characterized by increased uric acid and thiols groups production (18), suggesting the existence of a tight connection between dietary antioxidants and the redox network that counteracts dietary-induced oxidative/inflammatory stress. We showed that association of antioxidant-rich foods or juices to HFM significantly reduced the endogenous antioxidant response (19–21). A role for probiotics in the recovery of the dysbiosed gut microbiota has been proposed, probiotic consumption was shown to recover the intestinal microbial structure in hyperlipidemia (22). For these reasons, we postulate that probiotics could mitigate oxidative and inflammatory stresses also through the modulation of endogenous redox defenses in intestinal cells. For such purpose, we aimed to investigate the redox protective effects of *Lactobacillus Casei* Shirota on the cellular damages induced by an oxidative stressor in the enterocyte-like cell line TC7/human colon carcinoma cell line (Caco-2).

## Materials and Methods

### Epithelial Cell Culture

The human intestinal Caco-2/TC7 cell line was kindly provided by Monique Rousset (Institute National de la Santé et de la Recherche Médicale, INSERM, France). These cells derive from parental Caco-2 cells at late passage, exhibit a more homogeneous expression of differentiation traits and have been reported to express higher metabolic, and transport activities than the original cell line, more closely resembling small intestinal enterocytes (23). The cells were routinely maintained at 37°C in an atmosphere of 5% CO_2_/95% air at 90% relative humidity and used between passages 100 and 105 on plastic tissue culture flasks (75 cm^2^ growth area, Becton Dickinson, Milan, Italy) in Dulbecco’s modified minimum essential medium (DMEM; 3.7 g/L NaHCO_3_, 4 mM glutamine, 10% heat inactivated fetal calf serum, 1% non-essential amino acids, 10^5^ U/L penicillin, and 100 mg/L streptomycin). All cell culture reagents were from Euroclone (Milan, Italy). For the experiments, the cells were seeded on transwell filters (polyethylene terephthalate filter inserts for cell culture; Becton Dickinson) of 12 mm diameter, 0.45 µm pore size, as described below. After confluency, cells were left for 17–21 days to allow differentiation (24). Medium was changed three times a week.

### Bacterial Growth

*Lactobacillus casei* Shirota was isolated from commercial fermented milk. One milliliter of fermented milk was centrifuged at 800 *g* for 5 min at 4°C, the pellet washed twice in PBS and re-suspended in 15 mL of DeMan Rogosa Sharp (MRS) medium (DIFCO, Milan) and grown at 37°C under anaerobic conditions over night. Aliquots (1 mL) of stationary phase culture were used to prepare glycerols. Before the experiments, one glycerol was thawed in 15 mL of MRS and grown at 37°C under anaerobic conditions. After overnight incubation, bacteria were diluted 1:15 in fresh MRS and grown until mid-log phase. Bacterial cells were then harvested by centrifugation at 3,000 *g* for 10 min at 4°C and re-suspended in antibiotic- and serum-free DMEM w/o phenol red. Bacterial cell concentrations were determined in preliminary experiments by densitometry and confirmed by serial dilutions followed by CFU counts of *LS* on MRS agar after 48 h of incubation at 37°C under anaerobic conditions. The viability of LS grown on DMEM did not differ from that of bacteria grown on LB or MRS media, as tested in preliminary experiments by CFU counts after agar plating of bacterial inoculum from the different media.

### Cells Treatment

Differentiated Caco-2/TC7 cells grown on transwell filters were untreated or pretreated with 1 mL of DMEM containing 5 × 10^7^/CFU of LS for 1.5 h in the apical compartment. After pretreatment with LS, cells were treated or not with 20 mg/mL of 2,2′-Azobis (2-amidinopropane) dihydrochloride (AAPH; SIGMA) in order to induce oxidative stress for 2.5 h at 37°C. After treatment, cells were washed three times with PBS containing Ca^++^ and Mg^++^ and used for further analyses.

### Cell Permeability

Membrane barrier permeability of cells was assayed by measuring the transepithelial electrical resistance (TEER), according to Ferruzza et al. (25). TEER was monitored every day until differentiation to test the effects of the different treatments, using a Millicell Electrical Resistance system (Millipore). TEER was expressed as Ohm (resistance) × cm^2^ (surface area of the monolayer) after subtracting the filter resistance value. The TEER was checked before each experiment, and only cell monolayers with TEER >1,000 Ωcm^2^ were used. During the experiments, TEERs were followed each hour after AAPH treatment. Cell permeability was measured at the end of cell treatment by phenol red passage, according to Ferruzza et al. (26). Briefly, following three washes with PBS containing Ca^++^ and Mg^++^, 0.5 mL of 1 mM phenol red was added on cell monolayers in the apical (AP) compartment, whereas 1 mL of PBS was added in the basolateral (BL) compartment. After 1 h of incubation at 37°C, 0.9 mL of BL medium was collected, treated with 0.1 mL of 0.1 N NaOH, and read at 560 nm to determine the phenol red concentration (Tecan Infinite M200 microplate reader, Tecan Italia, Milan, Italy). The phenol red passage was expressed as apparent permeability (P app), as previously described (26). The tight junctions (TJs) were considered open when the apparent permeability of phenol red was ≥1 × 10^−6^ cm × s^−1^.

### Localization of TJ (ZO-1 and Occludin) and AJ (E-Cadherin and β-Catenin) Proteins

The protective effects of LS on membrane damages induced by oxidative stress were assessed evaluating tight and adherent junctions’ (AJs) principal proteins immunolocalization. Briefly, Caco-2/TC7 cells were washed three times with cold PBS containing Ca^++^ and Mg^++^, fixed in ice-cold methanol for 3 min, and then incubated with rabbit polyclonal anti-ZO-1, mouse monoclonal anti-occludin, mouse monoclonal anti-β-catenin, or rabbit polyclonal anti-E-cadherin antibodies (Zymed Laboratories), for 1 h. For secondary detection, the cells were incubated with fluorescein isothiocyanate (FITC) or tetramethylrhodamine isothiocyanate (TRITC) conjugated secondary antibodies (Jackson Immunoresearch, Milan, Italy), for 1 h. Stained monolayers were mounted on glass slides using the Prolong Gold antifade reagent (Molecular Probes, Invitrogen, Milan, Italy) and analyzed using a fluorescence microscope (Zeiss, Jena, Germany).

### Morphological and Ultrastructural Analysis (Scanning Electron Microscope) of Epithelial Co-Culture Caco-2/Tc7 and *Lactobacillus* Shirota

In order to evaluate the cytological and ultrastructural cellular structure, analytical examination by scanning electron microscopy was performed. Caco-2 cells were fixed in 10% formaldehyde (prepared from paraformaldehyde) with 2.5% glutaraldehyde for 12 h and dehydrated using an ascending scale of alcohols. Samples were treated by Critical Point Drying (Incorporating, Ashford, Kent England), using for the final dehydration anhydrite carbon dioxide as transition fluid. Dehydrated samples were fixed on supports for electronic microscopy scanning and spattered for 120 s with gold using a 30 mA current in the presence of an atmosphere of argon 0.2% (Quorum Q150RS, Technologies Ashford, Kent, England). Monolayer culture samples were observed using a SEM microscope EVO LS10 C. Zeiss (Assing).

### Western Blot Assay

Human colon carcinoma cell line/TC7 cells differentiated on transwell filters (1 × 10^6^ cells/filter) were untreated (control) or apically pretreated with 1 mL of medium containing LS (5 × 10^7^ CFU/mL) for 1.5 h before the addition of AAPH (20 mg/mL) for 2.5 h. *L. casei* Shirota and AAPH concentrations and incubation times were chosen based on preliminary experiments, in order to allow triggering of the inflammation pathway without disrupting the cell monolayer. After treatments, Caco-2/TC7 cell were washed three times with cold PBS to eliminate the medium and non-adherent bacteria, and lysed or homogenized, respectively, in cold radioimmunoprotein assay buffer (RIPA: 20 mM Tris–HCl pH 7.5, 150 mM NaCl, 0.1% SDS, 1% Na deoxycholate, 1% Triton X-100) supplemented with 1 mM phenylmethylsulphonyl fluoride, and protease inhibitor (Complete Mini, Roche, Milan) and phosphatase inhibitor cocktails (PhosSTOP, Roche). Cell lysates (50 µg total proteins), were dissolved in sample buffer (50 mM Tris–HCl, pH 6.8, 2% SDS, 10% glycerol, 100 g/L bromophenol blue, 10 mM beta-mercaptoethanol), heated for 5 min, fractionated by SDS-polyacrylamide gel (4–20% gradient) electrophoresis, and transferred to 0.2 mm nitrocellulose filters (Trans-Blot Turbo, Biorad, Milan). Membranes were incubated with the following primary antibodies: rabbit polyclonal anti-human NF-κB p65, P-p65, Nrf2, kelch-like ECH-associated protein 1 (Keap1), from Cell Signaling Technology (Danvers, MA, USA), mouse monoclonal α-tubulin, gastro-intestinal glutathione peroxidase (GPX2) (R&D System, Milan), or phospho S40 Nrf2 rabbit monoclonal antibodies (Abcam, Milan). Proteins were detected with horseradish peroxidase-conjugated secondary antibodies (Cell Signaling Technology) and enhanced chemiluminescence reagent (ECL kit LiteAblot Extend, Euroclone), followed by analysis of chemiluminescence with the charge-coupled device camera detection system Las4000 Image Quant (GE Health Care Europe GmbH, Milan). Relative levels of GPX2 were normalized to human α-tubulin that was not affected by treatments (data not shown), while the expression of P-p65 and P-Nrf2 proteins were normalized to their corresponding un-phosphorylated forms.

### P-p65 and P-Nrf2 Immunolocalization in Intestinal Cells

Human colon carcinoma cell line/TC7 cells differentiated on transwell filters (1 × 10^6^ cells/filter), were untreated (control) or apically pretreated with 1 mL of medium containing LS (5 × 10^7^ CFU/mL), for 1.5 h before adding AAPH (20 mg/mL) for 2.5 h. At the end of treatments, cells were washed with PBS and fixed in ice-cold methanol for 3 min. Localizations of P-p65 and P-Nrf2 were determined as follows. Briefly, cells were treated with rabbit polyclonal anti- P-Nrf2 or P-p65 antibodies (Cell Signaling Technology). For secondary detection, cells were incubated with TRITC-conjugated goat anti-rabbit IgG for P-p65 or with FITC-conjugated goat anti-rabbit IgG for P-Nrf2, and the cell nuclei were stained with 300 nM DAPI, added directly to the mounting medium. Stained monolayers were mounted on glass slides by using Prolong Gold antifade Reagent (Molecular Probes, Invitrogen, Milan) and analyzed under a confocal microscope (LSM 700, Zeiss, Jena, Germany).

### Measurement of Intracellular ROS Levels

The protective effect of LS on the accumulation of intracellular ROS was detected by flow cytometry using the peroxide-sensitive fluorescent probe 2′,7′-dichlorofluorescein diacetate (DCFH-DA) (Sigma-Aldrich, Milan). ROS levels were evaluated with the use of a non-fluorescent probe (DCFH-DA) that once inside the cell becomes deacetylated by cellular esterases yielding the non-fluorescent DCFH. When ROS are present, DCFH is oxidized into the highly fluorescent DCF. The assay was performed according to Georgantzopoulou et al. (27) with some modifications and it was optimized for the DCFH-DA concentration and the exposure time. Differentiated Caco-2/TC7 cells were seeded in 12-well transwell plates at 1 × 10^6^ cells and treated as described before. After treatments, cells were washed with PBS three times and incubated with 10 µM DCFH-DA in Hank’s balanced salt solution (CaCl_2_ 1.26 mM, MgCl_2_ 0.5 mM, MgSO_4_ 0.41 mM, KCl 5.33 mM, KH_2_PO_4_ 0.44 mM, NaHCO_3_ 4.0 mM, NaCl 138 mM, Na_2_HPO_4_ 0.34 mM, d-glucose 5.6 mM) at 37°C for 30 min in the dark. Cells were detached from filters using a trypsin/EDTA solution for 5 min at 37°C in dark. Supernatants were centrifuged and pellets washed in PBS and re-suspended with 500 µL of FACSFlow (Becton and Dickinson, Milan). Flow cytometry analysis was performed using a FACSCalibur flow cytometer (BD Biosciences). In order to exclude dead/dying cells and, therefore, non-specific antibody-binding cells, cells were gated according to forward and side scatter. At least 10,000 events were acquired and analyzed. Data were analyzed at 488 nm using CellQuest software (BD Biosciences).

Accumulation of intracellular ROS was also detected *in situ*, by fluorescence microscopy (Zeiss, Jena, Germany). Briefly, Intracellular ROS levels were measured using DCFH-DA (10 µmol/L) at 37.0°C for 1 h in dark. Stained monolayers were mounted on glass slides by using Prolong Gold antifade Reagent (Molecular Probes, Invitrogen, Milan, Italy) added with 300 nM DAPI. Cell images were captured with a fluorescence microscope (excitation and emission wavelengths were 488 and 520 nm, respectively). At least 10 fields for each slide were observed and green positive cells (ROS positive) and the total number of cells (nuclei/field) were measured. Results were expressed as percentage of redox positive cell.

#### RNA Extraction and RT-qPCR Experiments

Total RNA from Caco-2 cells was extracted using the RNeasy Plus Mini Kit (Qiagen, Hilden, Germany), according to the manufacturer’s instructions. Purity, integrity, and concentration were analyzed using the Agilent 2100 Bioanalyzer (Agilent Technologies, Milan, Italy). RNA was reverse transcribed and amplified using the Power SYBR Green RNA-to-CT 1-Step Kit (Life Technologies, Monza, Italy) according to manufacturer’s instructions and applying the following thermal protocol: reverse transcription for 30 min at 48°C; Taq activation for 10 min at 95°C; 40 cycles of denaturation for 15 s at 95°C, and annealing/extension for 1 min at 60°C. Finally, melting curve analysis was performed in order to verify the proper product amplification. Optimal input RNA concentration for each gene was chosen using standard curve analysis of a pool of RNA samples. The human actin gene was selected as an internal standard. Primers were QuantiTect Primer Assay from Qiagen (Cat. numbers: actin, beta: QT01680476; GPX2: QT00200039). Measurements were performed in technical triplicate and repeated at least three separate times. Data in figures are expressed in Log_2_ in order to provide symmetrical distribution of gene expression effects and reported as percentage of gene expression in control cells. All data are expressed as the ratio to the reference gene Actin.

### Statistical Analysis

Statistical significance of the differences was evaluated by one-way ANOVA followed by *post hoc* Tukey HSD test after normality test analysis. For gene expression, the unpaired Student’s *t*-test was used. The significance was set at *P* values <0.05. In the figures, * and ** represent *P* values <0.05 and <0.01, respectively. The symbol * was used to indicate significant difference from the control group. All statistical analyses were performed with the software program “Statistica” (version 5.0; StatSoftInc., Tulsa, OK, USA).

## Results

### Membrane Barrier Permeability

**Figure 1:**
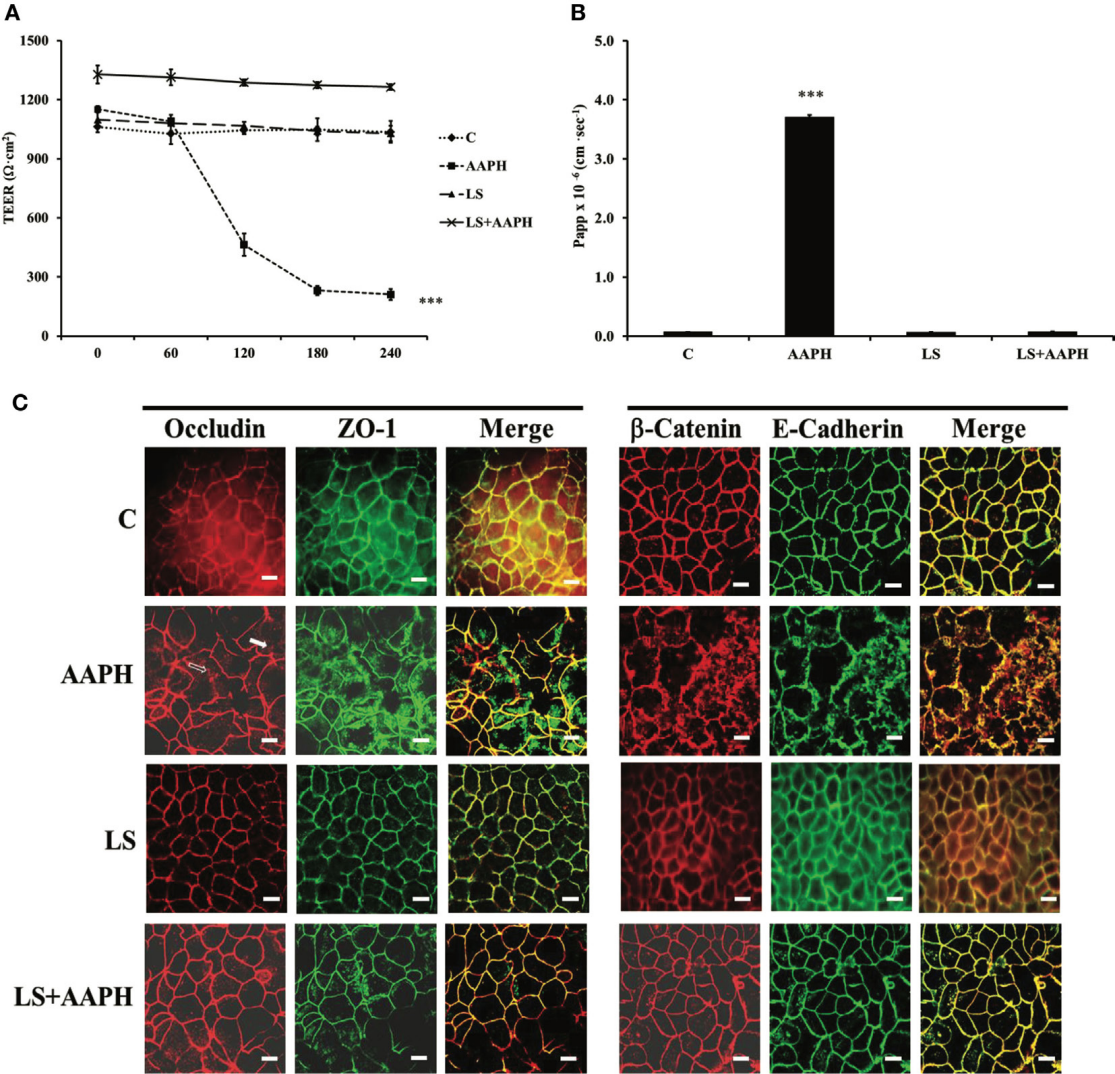
Effect on barrier integrity: protective effect of *Lactobacillus casei* Shirota (LS) in inhibiting the increased membrane permeability caused by AAPH in human colon carcinoma cell line cells. Cells differentiated on transwell filters were untreated (C), treated with AAPH (AAPH), treated with LS or pretreated with LS for 1.5 h, and then treated with AAPH (LS + AAPH) for 2.5h. Transepithelial electrical resistance [TEER, **(A)**] was measured every 60 min, from 0 to 240 min and reported as Ohm × cm^2^. After treatments, phenol red was added in the apical compartment, for 1 h, and then determined spectrophotometrically in the basolateral compartment. The results are expressed as apparent permeability [Papp, **(B)**]. Values represent mean ± SD of at least three independent experiments, carried out in triplicate. *** Statistically different from control cells (*P* < 0.001). Cells were labeled with specific primary antibodies for tight (ZO-1 and occludin) and adherent (β-catenin and E-cadherin) junction proteins **(C)**, followed by tetramethylrhodamine isothiocyanate (TRITC)- and fluorescein isothiocyanate (FITC)-conjugated secondary antibodies for occludin and ZO-1 and for β-catenin and E-cadherin, respectively. As shown, AAPH treatment determined the loss of the membranous continuous staining of ZO-1, occludin, β-catenin, and E-cadherin. Dissociation of occludin from membrane is indicated by arrows. Regular localization of tight and adherent junction proteins is visible in cells treated with LS alone or pretreated with LS before AAPH treatment. Each figure is representative of three independent immunofluorescence assays (63× magnification). Bars represent 10 µm.

In order to test if LS had protection capabilities toward AAPH-induced cell damages in Caco-2/TC7 cells, cell permeability and TJ and AJ protein junctions were analyzed. Membrane barrier permeability was assessed through the evaluation of the TEER and the paracellular flux of the phenol red marker, are shown in Figures 1A,B, respectively. Neither control nor LS-treated cells displayed any significant change in TEER values throughout the entire incubation time, with respect to T0 cells. On the other hand, AAPH treatment induced a drastic and significant drop of TEER, starting within 60 min (***P < 0.001), with respect to T0 (Figure 1A). TEER drop induced by AAPH was totally prevented in cells pretreated with LS. AAPH effects were further confirmed using the paracellular flux of the phenol red (Figure 1B), the increase of which (***P < 0.001) was fully reversed by LS pretreatment. Cells treated with LS alone did not exhibit any changes in the paracellular flux.

### Localization of ZO-1, Occludin, E-Cadherin, and β-Catenin

Protective effects of LS on Caco-2/TC7 cells protein junctions were analyzed by immune fluorescence. The immunolocalization of TJ proteins in Caco-2/TC7 cells is shown in Figure 1C. As shown, LS treatment did not modify ZO-1 and occludin localization around cell boundaries. On the other hand, treatment with AAPH caused ZO-1 and occludin delocalization from the membrane, with a scattered distribution of ZO-1 inside the cells and partially loss of occludin from cell membrane (empty arrow) associated with translocation inside the cells (white arrow), a marker of TJ disruption. Pretreatment with LS protected the cell membrane, as highlighted by the correct distribution and organization of TJ proteins. Similar effects were observed on the AJ proteins E-cadherin and β-catenin: while treatment with LS alone had no effects, AAPH was able to induce severe architectural changes of both AJ proteins, as shown by irregular staining. On the other hand, this displacement of AJ proteins from the cell–cell junctions was completely counteracted by LS pretreatment.

### Inhibition of AAPH-Induced Inflammatory Status Activation by LS

**Figure 2:**
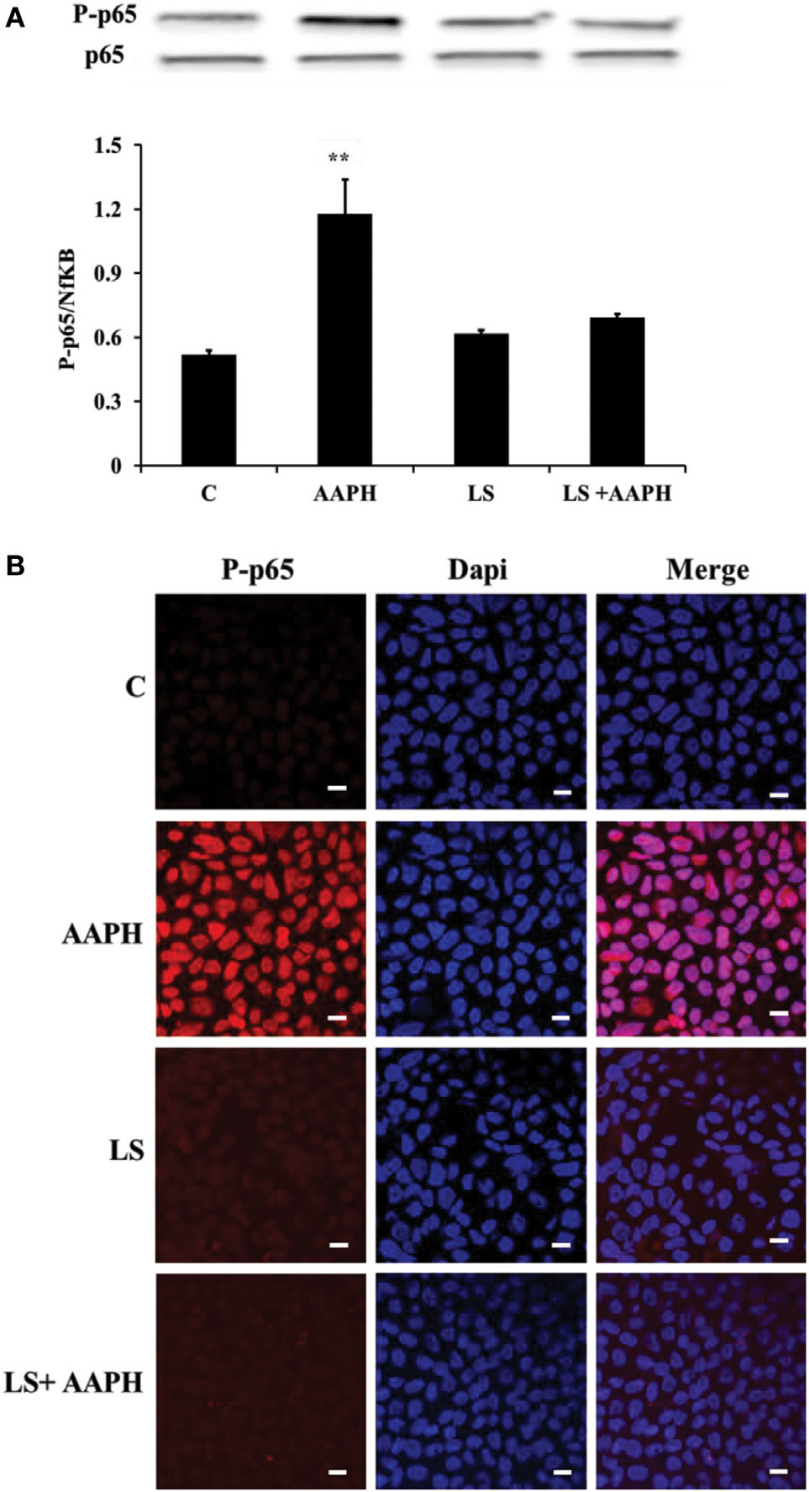
Inhibition of AAPH-induced inflammatory signaling pathways by *Lactobacillus casei* Shirota (LS) in human colon carcinoma cell line/TC7 cells. Cells differentiated on transwell filters were untreated (C), treated with AAPH (AAPH) treated with LS or pretreated with LS for 1.5 h, and then treated with AAPH (LS + AAPH) for 2.5 h. Western blot densitometric values of phosphorylated p65 were normalized with un-phosphorylated p65 **(A)**. Values represent mean ± SD of three independent experiments, carried out in triplicate. **P < 0.01 immunolocalization of P-p65 **(B)** was performed by confocal microscopy, in order to evaluate its translocation into the nucleus. For P-p65 immunofluorescence analysis, cells were labeled with specific primary antibodies against P-p65 and subsequently with tetramethylrhodamine isothiocyanate secondary antibodies, while nuclei were stained in blue (DAPI). Each figure is representative of three independent assays (63× magnification).

To investigate the possibility that LS could inhibit transmission of redox cascade in Caco-2/TC7 cells following AAPH treatment, the phosphorylation status of p65 was assessed by means of Western blot analysis. Results indicate that treatment with AAPH induced a significant increase of phosphorylation of the p65 subunit (**P < 0.01), compared to the phosphorylation level of p65 observed into control Caco-2/TC7 cells, the effect was completely prevented when the cells were pretreated with LS (Figure 2A). Pretreatment with LS did not induce any changes in p65 phosphorylation, indicating that probiotic alone does not induce Nf-κB migration into the nucleus. Immunolocalization analysis of P-p65 strengthens the results obtained by Western blot (Figure 2B): nuclear localization of P-p65 was detected only in cells treated with AAPH but not in cells pretreated or treated only with LS.

### Effect of LS on Induction of Nrf2 Pathway

**Figure 3:**
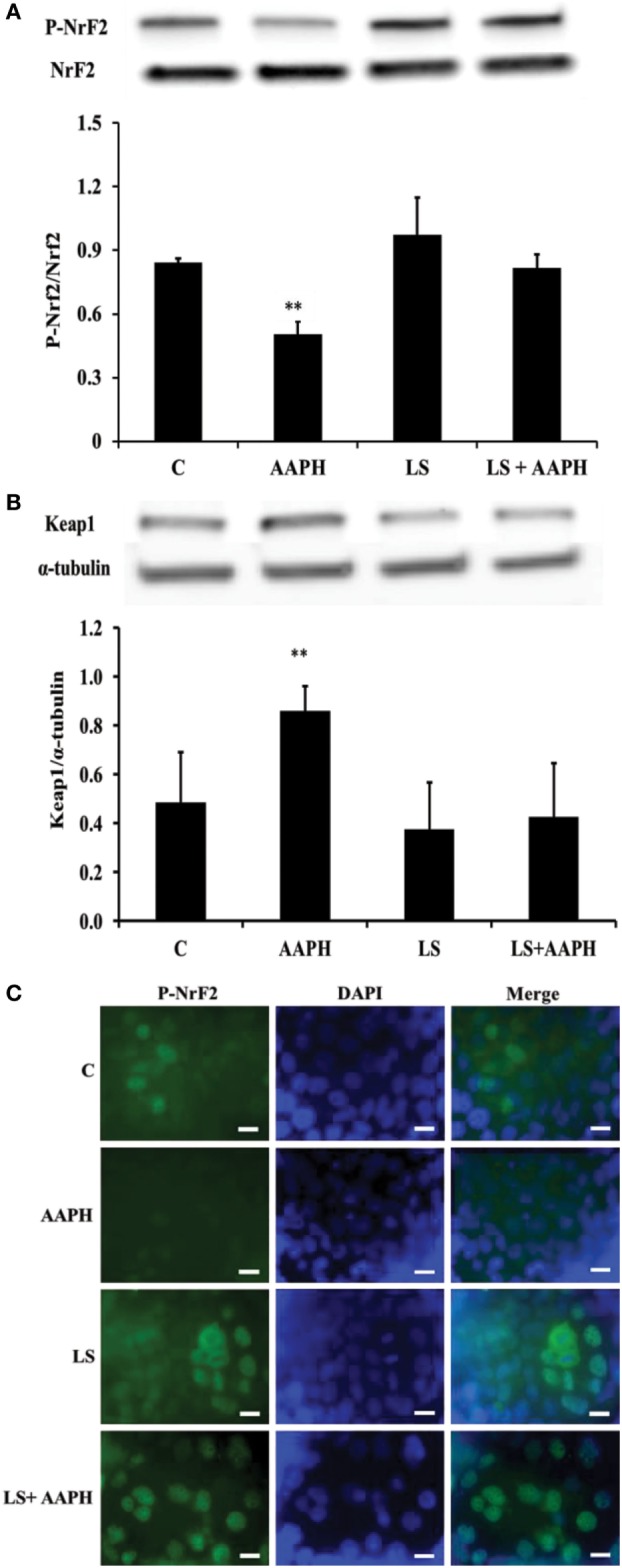
Effect on nuclear factor erythroid 2-related factor 2 (Nrf2) regulation of *Lactobacillus casei* Shirota (LS) in human colon carcinoma cell line/TC7 cells treated with reactive oxygen species inducer AAPH. Western blot densitometric values of normalized P-Nrf2 and of α-tubulin-normalized kelch-like ECH-associated protein 1 protein levels **(A,B)**. Values represent mean ± SD of three independent experiments, carried out in triplicate. *P < 0.05 compared with all. Immunolocalization of P-Nrf2 **(C)** was performed by confocal microscopy in order to evaluate translocation into the nucleus. For P-Nrf2 immunofluorescent analysis, cells were labeled with specific primary antibodies against P-Nrf2 and subsequently with FITC secondary antibodies, while nuclei were stained in blue (DAPI). Each figure is representative of three independent assays (100× magnification). Bars represent 10 µm.

To investigate the relationship between inflammation and the antioxidant response in Caco-2/TC7 cells, we analyzed the phosphorylation of Nrf2, the expression of Keap1 and nuclear localization of P-Nrf2. We found that treatment of Caco-2/TC7 cells with AAPH induced a significant reduction of Nrf2 phosphorylation (**P < 0.01) and that LS-pretreatment maintained p-Nrf2 cellular levels similar to that of control and LS-treated cells (Figure 3A). On the other hand, the expression of Keap1 resulted significantly increased in Caco-2/TC7 cells treated with AAPH (**P < 0.01) but not in LS-treated cells nor in LS + AAPH-treated cells (Figure 3B). Immunolocalization of P-Nrf2 supported Western blot data (Figure 3C) showing the nuclear localization of P-Nrf2 upon pretreatment with LS and absence of P-Nrf2 in Caco-2/TC7 cells treated with AAPH alone. Slide field-quantification of P-Nrf2 positive cells indicates that LS induced a significant increase of P-Nrf2 positive cells (53.6% ± 6.0) also after AAPH treatment (58.5% ± 7.6), compared to C (25.7% ± 4.7), and AAPH-treated cells (21.3% ± 4.3) (*P* < 0.01).

### Cellular Redox Status (DCFH Assay)

**Figure 4:**
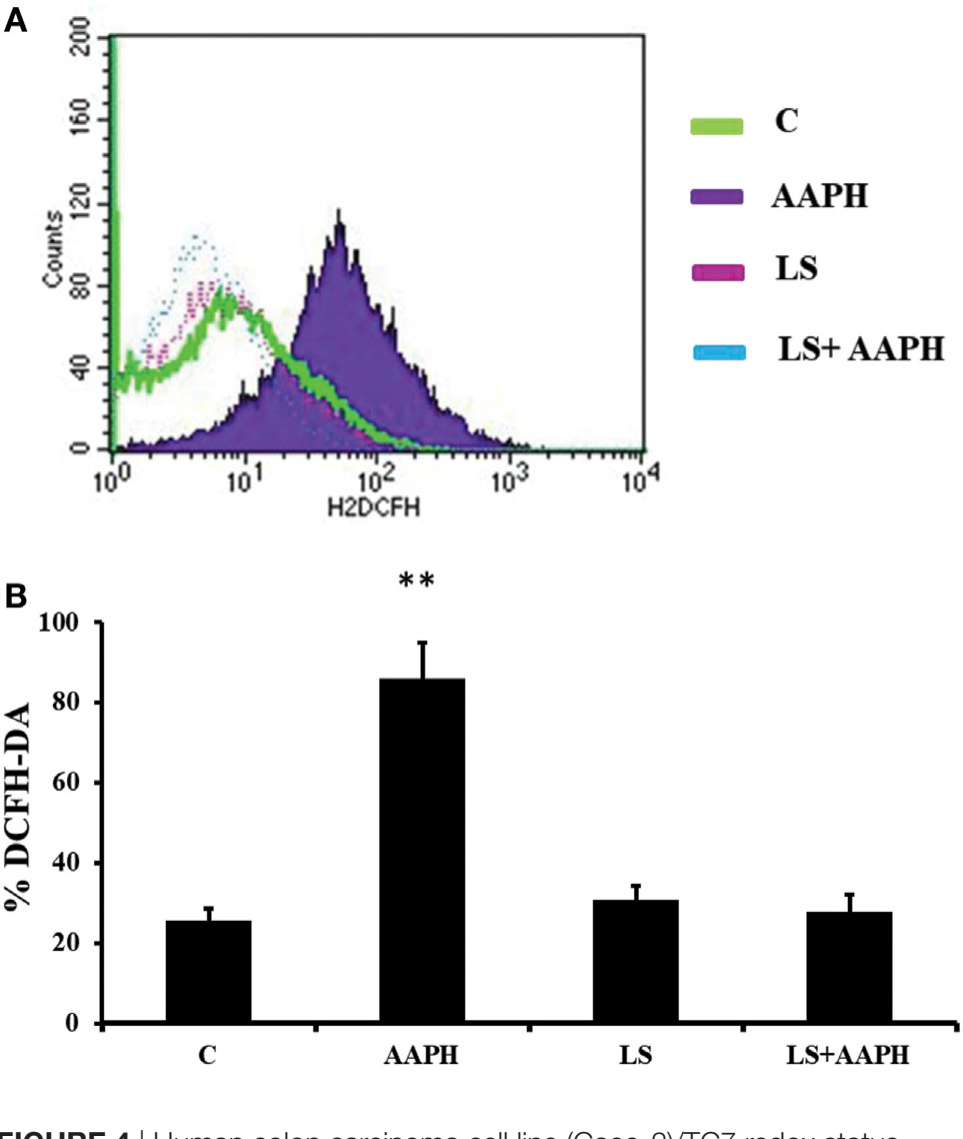
Human colon carcinoma cell line (Caco-2)/TC7 redox status. The accumulation of intracellular reactive oxygen species (ROS) was detected using the peroxide-sensitive fluorescent probe 2′,7′-dichlorofluorescein diacetate (DCFH-DA). Cells differentiated on transwell filters were untreated (C), treated with AAPH treated with *Lactobacillus casei* Shirota (LS) or pretreated with LS for 1.5 h and then treated with AAPH (LS + AAPH) for 2.5 h. After treatments, cells were washed three times with PBS and incubated with 10 µM DCFH-DA in HBSS. Redox status was measured by flow cytometry analysis **(A)** acquiring at least 10,000 events. The figure is representative of three independent assays. Fluorescence microscope analysis was performed on Caco-2/TC7 cells treated as previously described. The percentage of ROS positive cells with respect to total cells/field was calculated **(B)**. At least 10 fields for each treatment were observed in three independent immunofluorescence assays. Data are reported as mean ± SD of percentage of DCFH-DA positive cells. Differences with *P* values <0.05 were considered significant and indicated within each parameter by **P < 0.01 vs C.

In order to confirm the generation of ROS by AAPH in Caco-2/TC7 cells and the effect of LS on cellular redox status, DCFH was tested by flow cytometry analysis and immunofluorescence. Flow cytometry analysis (Figure 4A) indicates that AAPH-treated Caco-2/TC7 cells contains higher amount of ROS as shown by the shift of fluorescence that was not present in Caco-2/TC7 cells pretreated with LS. These results were confirmed by microscope analysis of fluorescence showing that cells pretreated with LS displayed a drastic reduction of the fluorescence intensity induced by AAPH. The pretreatment with LS alone did not induce any changes compared to control cells. In order to quantify the effect of LS on oxidative stress induced by AAPH, the number of DCFH-DA positive cells was described as the percentage of DCFH-DA positive cells on the total number of cells/field (Figure 4B). The results show that Caco-2/TC7 cells treated with AAPH were almost completely positive (86.3% ± 9.1%), differently from all other treatments and control (*P* < 0.01), whereas the number of positive cells present in LS-treated cells (30.7% ± 3.5%) or LS plus AAPH (27.8% ± 4.5%) was not significantly different to the number observed in the control (25.6% ± 3%).

### Intestinal Glutathione Peroxidase Gene/Protein Expression

**Figure 5:**
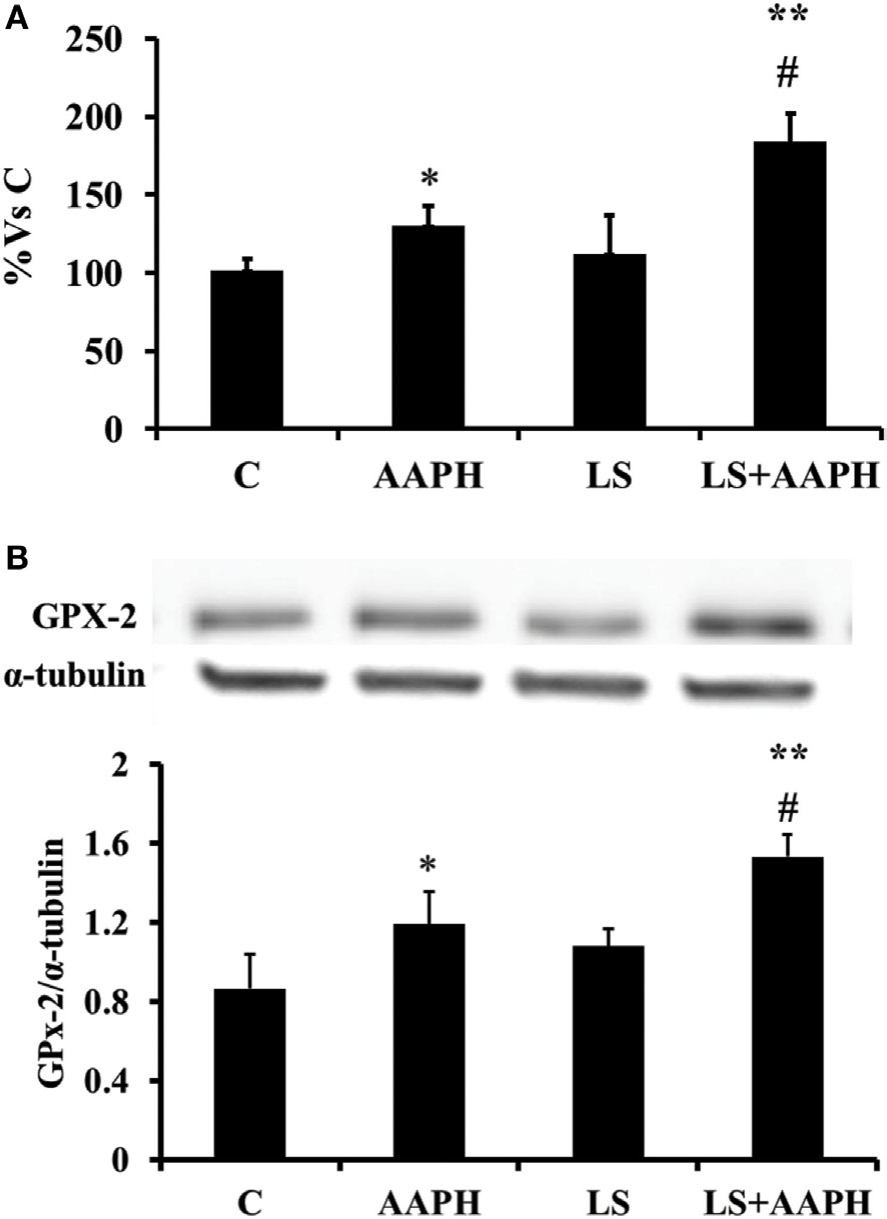
Effect of *Lactobacillus casei* Shirota (LS) on expression of GPX2 in human colon carcinoma cell line/TC7 cells. Gene expression of gastrointestinal glutathione peroxidase was evaluated by real-time PCR. Cells differentiated on transwell filters were untreated (control), treated with AAPH (AAPH) treated with LS (LS) or pretreated with LS for 1.5 h, and then treated with AAPH (LS + AAPH) for 2.5 h. After treatments, the cells were washed and total RNA extracted. Gene expression of GPX2 **(A)** was performed in technical triplicate and repeated at least three separate times. (Data in figures are expressed in Log_2_ in order to provide symmetrical distribution of gene expression effects and reported as percentage of gene expression in control cells). All data are expressed as the ratio to the reference gene Actin. Statistical significance was determined by unpaired Student’s *t*-test. Differences with *P* values <0.05 were considered significant and indicated within each parameter by *P < 0.05 vs C, **P < 0.01 vs C, and ^#^*P* < 0.01 vs AAPH. Protein expression was evaluated by Western Blot **(B)**. Cells differentiated on transwell filters were untreated (control), treated with AAPH (AAPH) treated with LS or pretreated with LS for 1.5 h, and then treated with AAPH (LS + AAPH) for 2.5 h. After the treatments, the cells were lysed and fractionated by SDS-PAGE and transferred to nitrocellulose filters. The membranes were incubated with rabbit anti-gastro-intestinal glutathione peroxidase and anti-α-tubulin primary antibodies, and then with horseradish peroxidase-conjugated secondary antibodies. The figure shows a representative Western blot of GPX2 (upper panel) and the densitometric values of the immunoreactive protein bands (lower panel). The relative expression levels of GPX2 were normalized to α-tubulin levels. Values represent mean ± SD of at least three independent experiments, carried out in triplicate. Differences with *P* values <0.05 were considered significant and indicated within each parameter by *P < 0.05 vs C, **P < 0.01 vs C, and ^#^*P* < 0.01 vs AAPH.

In order to check if the modulatory effects of LS observed on Nrf2 activation could involve an activation of antioxidant enzymes at gene expression level, mRNA levels were quantified by means of real-time qPCR. Data are reported as percentage of control that was considered 100%. Gene expression of gastrointestinal GPX2 in Caco-2/TC7 cells (Figure 5A) treated with AAPH was slightly, but significantly higher compared to control cells (*P < 0.05) whereas a strong increase of GPX2 expression was observed when the cells were pretreated with LS before AAPH treatment (**P < 0.01). In order to evaluate the effects at the protein level we then analyzed protein expression of GPX2 by Western blot (Figure 5B). At protein level, GPX2 expression shows a slight but significant induction in cells treated with AAPH compared to control cells (*P < 0.05). Caco-2/TC7 cells pretreated with LS and AAPH showed a significant increase of GPX2 protein expression compared to control (**P < 0.01) and AAPH treatment (*P < 0.05). Significant difference was observed between cell treated with AAPH alone or LS + AAPH (^#^*P* < 0.05), whereas no differences were observed in cells treated with LS.

### Morphological and Ultrastructural Analysis (Scanning Electron Microscopy) of Epithelial Co-Culture Tc7 and *L. casei* Shirota

**Figure 6:**
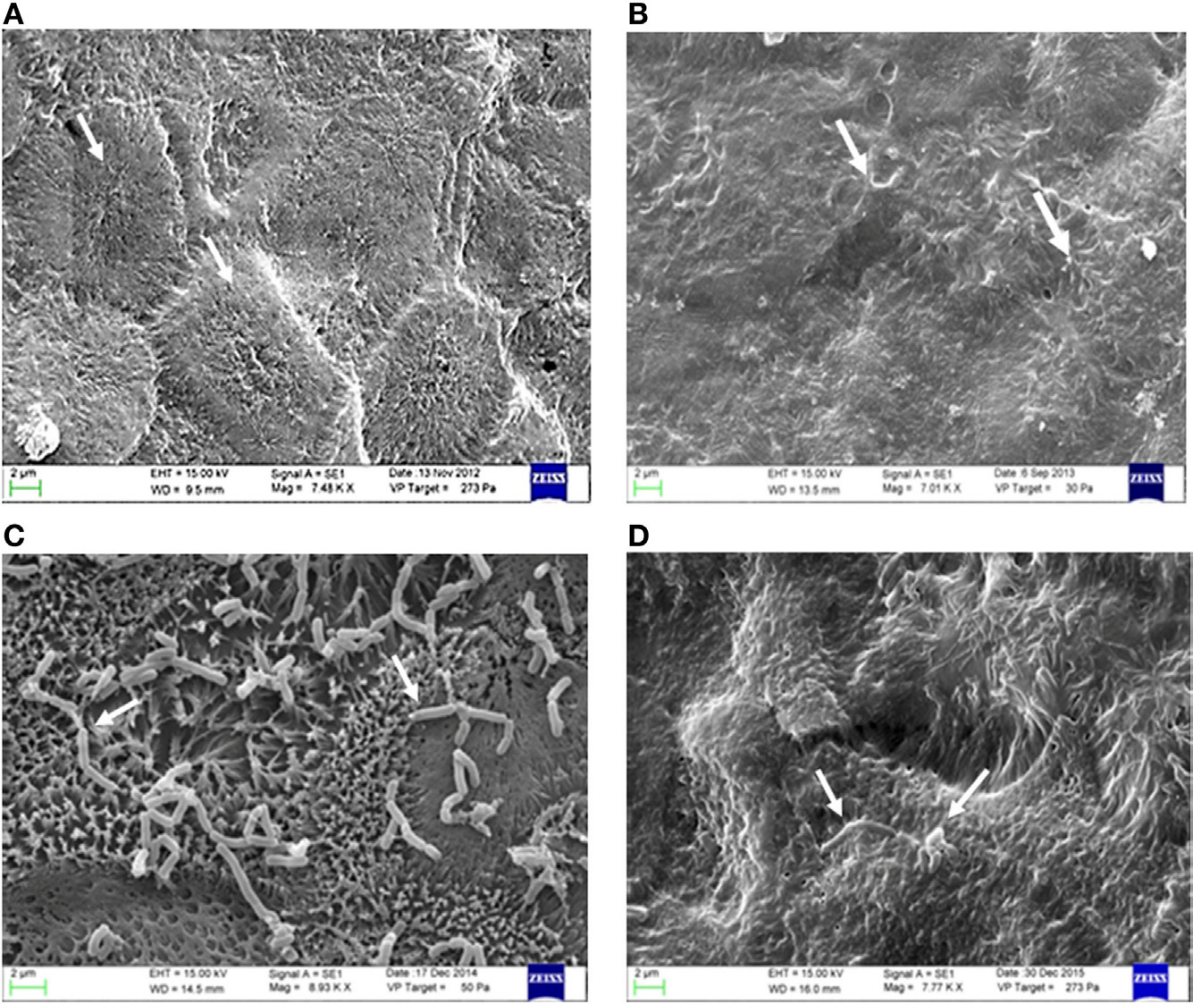
Morphological and ultrastructural analysis (electron microscopy) of epithelial co-culture Tc7 and *Lactobacillus casei* Shirota (LS). The **(A)** show the morphological state of the monolayer epithelium consisting of the human colon carcinoma cell line (Caco-2)/Tc7 culture cell line in physiological equilibrium (control). The **(B)** show the differentiated Caco-2/TC7 cells grown on transwell filters and treated with 20 mg/mL of 2,2′-Azobis (2-amidinopropane) dihydrochloride (AAPH) for 2.5 h at 37°C. The arrows indicate that the cell margins are not evident, suggesting a possible cellular membrane damage. Panel **(C)** shows the monolayer co-culture of the Caco-2/tc7 pretreated with 1 mL of DMEM containing 5 × 10^7^/CFU of LS for 1.5 h in the apical compartment. Panel **(D)** shows morphology of the Caco-2/TC7 cells pretreated with 1 mL of DMEM containing 5 × 10^7^/CFU of LS for 1.5 h in the apical compartment. After pretreatment with LS, cells were treated with 20 mg/mL of AAPH in order to induce oxidative stress for other 2.5 h at 37°C. The arrows indicate a strong discrasia of the LS cells which has various forms and different size. The figure is representative of at least three experiments conducted in triplicate.

In order to corroborate protein and gene findings also at the morphological level, a cytological and ultrastructural cellular structure analysis of Caco-2/TC7 cells by means of scanning electron microscopy was performed. Figure 6A shows the morphological state of the monolayer epithelium consisting of the Caco-2/Tc7 cells in physiological equilibrium (control). A normal cellular morphology is observed, with a well-established brush border with well-defined and aligned apical microvilli, demonstrating correct cell differentiation. In particular, the cellular margins appear sharp and marked, with the typical morphological mosaicism of *in vivo* intestinal epithelium. The morphological state of the monolayer epithelium following Caco-2-/-Tc7 treatment with AAPH is displayed in Figure 6B. As shown, under oxidative stress, the cell epithelium morphology changed drastically and the monolayer of the Caco-2/Tc7 cell line switched to a less defined aspect compared to the control, with less evident cells margins, clustering of apical microvilli in small pyramidal tufts with fusion of the apical portions. Figures 6C,D show the effects of LS preincubation. As shown, no appreciable structural changes were induced both in the cellular epithelium and in the cellular component, by LS. The figures show the close relationship between the two cellular components (eukaryotic and prokaryotic cells), which occurs predominantly in the microvilli apical area where the glycocalyx (glycoprotein-polysaccharide covering the superficial cell membranes) is plenty. In Figure 6D, showing Caco-2/Tc7 cells stressed with AAPH in presence of LS, a strong dyscrasia of LS is evident, with cells having various forms and size, with a significant numerical decrease compared to the co-culture in Figure 6C. The Caco-2/Tc7 monolayer epithelium in presence of LS cells displayed normal-like morphology compared to the corresponding epithelium observed in Figure 6B, demonstrating that LS incubation is able to prevent disruption of the Caco-2/Tc7 epithelium induced by AAPH.

## Discussion

Protective effects of probiotics have been well established on membrane barrier functions (2), immune functions (28), allergy (29), pathogen response (30), and inflammation, especially for intestinal chronic diseases improvement (31, 32). However, no data are available in the literature concerning their relationships with the mechanisms responsible for cellular redox signals.

In this study, we show that *in vitro* pretreatment of differentiated Caco-2/TC7 intestinal cells with LS probiotics reduces membrane barrier damages induced by AAPH-induced oxidative stress, and the effect is associated with the modulation of Nrf2/Keap1 signaling and with inhibition of the NF-κB inflammatory pathway. For the first time to our knowledge, we show that the protective effect of LS involves both non-enzymatic and enzymatic redox mechanisms, namely through the activation of antioxidant defenses and the modulation of GPX2 activity. LS inhibited in Caco-2/TC7 cells the AAPH-induced drop of the electrical resistance (TEER), and the increase of the paracellular passage of phenol red, which are markers of cellular membrane barrier damages associated with the disruption of cellular tight and AJs. Accordingly, by means of immunostaining we show that AAPH induced a severe impairment and delocalization of ZO-1, β-catenin, and E-cadherin, together with the loss of continuity around cell boundaries. Such results are in agreement with previous reports by Rao and coworkers, that reported phosphorylation of ZO-1, occludin, β-catenin, and E-cadherin on tyrosine residues following oxidative stress (33, 34), a key mechanism responsible for the delocalization of junctional proteins from cell boundaries. Importantly, LS pretreatment abolished the oxidative injury induced by AAPH on Caco-2/TC7 tight and AJs, preventing the TEER drop and the associated increase of membrane permeability. Tight and AJs are the major barrier components of epithelial monolayers and they maintain the epithelial barrier integrity and the apical-basolateral cell polarity. Recent evidence suggests that AJ and TJ proteins also participate in signal transduction mechanisms in epithelial cells such as occludin and proteins from the ZO family, that participate in the regulation of cell growth and proliferation (35, 36). ZO proteins distribution is known to be affected by inflammatory status and it is largely showed that inflammatory cytokines are able to induce structural and functional perturbations of TJ, that result to enhance paracellular permeability (37, 38). Phosphorylation of specific tyrosine residuals and other protein modification, such as thiol oxidation, nitration, and carbonylation, has been shown as the initial signal involved in the oxidative stress-induced disruption of TJs, through ROS, such as hydrogen peroxide, nitric oxide, peroxynitrite, and hypochlorous acid, in a process that results in the disruption of the morphology of cellular junctions and in the loss of the epithelial and endothelial barrier functions. Moreover, ROS may oxidize actin and tubulin causing the disassembly of F-actin and tubulin, which leads to cytoskeleton disruption and intestinal barrier dysfunctions (34, 39). As demonstrated by immunofluorescence staining, AAPH treatment induced in Caco-2/TC7 cells a high inflammatory status, i.e., increased nuclear localization of both p65 and Nrf2, and different effects on their phosphorylation levels, that were found up- and downregulated respectively. Flow cytometric analysis and fluorescent staining of ROS is consistent with the establishment by AAPH of an ROS-dependent inflammatory status in Caco-2/TC7 cells. Pretreatment with LS avoided ROS levels increase and Nrf2 dephosphorylation, thus preventing the establishment of the AAPH-induced inflammatory status. Together with NF-κB, Nrf2 is a transcription factor involved in the regulation of inflammatory pathways associated with oxidative stress. NF-κB is one of the major redox-sensitive transcription factors and is activated by ROS following the regulation of genes encoding proinflammatory mediators (40–42). Nrf2 is considered the primary cellular defense against oxidative stress-induced cytotoxicity, through the induction of phase II detoxifying (glutathione S-transferases) and antioxidant enzymes (GPX2, SOD, HO-1, and NQO1) (40, 42, 43). Under unstressed conditions, Nrf2 is repressed in the cytoplasm by the binding of the Keap1. Upon minimal stress conditions, Nrf2 is translocated into the nucleus where it binds to the antioxidant response element trans-activating a number of antioxidant genes (7, 44). According with our results, tight regulation of Nrf2 and NF-κB is necessary for the maintenance of the redox homeostasis in healthy cells, as previously demonstrated (42). Our observations are consistent with the report that upon intense oxidative stress, Nrf2 remains within the cytoplasm where it is antagonized by p65 through deprivation of the CREB binding protein (CBP), as reported by Liu and colleagues. The authors showed that in p65-overexpressing Hepg2 cells, the ARE-dependent expression of heme oxygenase-1 was strongly suppressed by two mechanisms. In the first one, p65 selectively deprives CBP from Nrf2 by a competitive interaction with the CH1-KIX domain of CBP, inducing the inactivation of Nrf2 that it is associated with the phosphorylation of p65 at S276. The second mechanism proposed involves recruitment of the histone deacetylase 3 (HDAC3) corepressor by p65 to the ARE, by facilitating the interaction of HDAC3 with either CBP or MafK, leading to local histone hypoacetylation (45).

In a recent study, Gao and colleagues (46) observed that treatment of high-fat fed mice with *Lactobacillus plantarum* FC225, was able to significantly increase the activities of free radical detoxifying enzymes, such as superoxidase dismutase and glutathione peroxidase, and decreased the content of malondialdehyde in liver homogenates. Furthermore, according with our results, it has been shown that some probiotic strains are able to induce nuclear migration of Nrf2 in intestinal porcine epithelial cells (47), in hepatocytes, and *in vivo* in rats (46, 48).

GPX2 catalyzes the reduction of organic hydroperoxides and hydrogen peroxide by glutathione in the intestine, protecting cells against oxidative damage, and together with other GPX2 provides a very important protection against inflammation and cancer (49). As showed by gene and protein expression, AAPH treatment induced a slight but significant increase of GPX2 compared to control cells, suggesting the activation of the antioxidant defense against radical charge. Notably, a strong increase of the GPX2 expression was observed also following LS treatment, indicating that the probiotic is able to promote a further improvement of the antioxidant defense in the presence of a free radical stimulus. This observation is consistent with the previously reported ability of lactobacilli to induce Nrf2-ARE and GPX2 expression. Such effect was observed both *in vitro* in mouse embryotic fibroblast and NIT-3T3 cells, using *Lactobacillus gasseri* or metabolites from *L. plantarum* on HepG2 cells, and *in vivo* in pig’s jejunum, using *Lactobacillus reuteri* (50–52). Another *in vivo* study conducted with diabetic patients demonstrated that consumption of yogurt containing probiotics (*Lactobacillus acidophilus* La5 and *Bifidobacterium lactis* Bb12) for 6 weeks was able to influence the oxidative status in type 2 diabetic patients, inducing decreased fasting blood glucose and glycated hemoglobin and increased activities of SOD and GPX2, and total antioxidant status compared with the control yogurt group without probiotics (53). Finally, increased SOD and GPx activities were reported also in high-fat fed mice following treatment with *L. plantarum* FC225 (46). According to the observed dephosphorylation of Nrf2 induced by AAPH, we cannot exclude that LS could induce GPX2 through a different, Nrf2-independent pathway. Accordingly, in a previous study in animals with DSS-induced colitis, Hiller and colleagues found that GPX2 was induced through activation of STAT3, a transcription factor involved in wound healing, proliferation, and protection from apoptosis during acute injury (54). Moreover, the authors found a strong reduction of GPX2 expression in STAT3 deleted lung cells even if Nrf2 KO in epithelial cells did not abolish completely GPX2 expression (54). We hypothesize that in our model AAPH induces an inflammatory injury that activates GPX2 expression in a Nrf2-independent manner, probably through the transcription factor STAT3, whereas *Lactobacillus* and its metabolites induce GPX2 transcription through the activation of the Nrf2 pathway. In any case, increased expression of GPX2 suggests that administration of probiotics promotes the development of some cellular antioxidant defense mechanisms against species generating ROS.

Morphological and ultrastructural analysis confirmed that LS is able to adhere to differentiated enterocyte-like Caco-2 cells. Morphological analysis indicates that AAPH treatment induced several damages to the epithelial monolayer as indicated by thinner cell margins and by the observation that microvilli tend to group in clumps clustered with the apical portions fused (Figure 6). We found that in cells pretreated with the probiotic, the damages induced by AAPH were not observed, indeed the epithelial monolayer showed a control cell-like morphology (Figure 6).

## Conclusion

We provide evidence that *L. casei* Shirota incubation prevents the loss of membrane integrity modulating inflammatory and oxidative stress induced by AAPH. The mechanism of the observed effect is related to the modulation of the Nrf2/Keap-1 signaling, involves the inhibition the NF-κB inflammatory pathway and an increase of cellular redox status and an activation of GPX2. Despite further evidences are needed, our results suggest a novel mechanism of action for probiotics, based on the modulation of cellular redox status.

## Author Contributions

AF and RA performed the literature search, data collection, cellular analysis, and wrote the first draft of the manuscript. AF, RA, and MS designed the experimental set-up and provided interpretation of the results. FN, IG, and AR performed SEM experiments and treatments. MS conceived the study and revised the manuscript.

## Conflict of Interest Statement

None of the authors have a competing financial interest in relation to the presented work.

## Abbreviations

AAPH, 2,2′-Azobis (2-amidinopropane) dihydrochloride; LS, *Lactobacillus casei Shirota*; AJs, adherent junction; TJs, tight junctions; GP-X, glutathione peroxidase; GPX2, gastro-intestinal glutathione peroxidase; Nrf2, nuclear factor erythroid 2-related factor 2; NF-κB, nuclear factor kappa B; Keap-1, Kelch-like ECH-associated protein 1 (Keap1); ARE, antioxidant response element; TEER, trans epithelial electrical resistance; Caco-2, human colon carcinoma cell line; ROS, reactive oxygen species; TNF-α, tumor necrosis factor-α; IL-, interleukin; DCFH-DA, dichloro-dihydro-fluorescein diacetate.
